# Mesenchymal Stem Cells and Transforming Growth Factor-β_3_ (TGF-β_3_) to Enhance the Regenerative Ability of an Albumin Scaffold in Full Thickness Wound Healing

**DOI:** 10.3390/jfb9040065

**Published:** 2018-11-14

**Authors:** Dale S. Feldman, John F. McCauley

**Affiliations:** Department of Biomedical Engineering, The University of Alabama at Birmingham, UAB, Birmingham, AL 25294, USA; 357dale@gmail.com

**Keywords:** mesenchymal stem cells, TGF-β_3_, albumin scaffold, wound healing, pressure ulcers

## Abstract

Pressure ulcers are one of the most common forms of skin injury, particularly in the spinal cord injured (SCI). Pressure ulcers are difficult to heal in this population requiring at least six months of bed rest. Surgical treatment (grafting) is the fastest recovery time, but it still requires six weeks of bed rest plus significant additional costs and a high recurrence rate. A significant clinical benefit would be obtained by speeding the healing rate of a non-surgical treatment to close to that of surgical treatment (approximately doubling of healing rate). Current non-surgical treatment is mostly inactive wound coverings. The goal of this project was to look at the feasibility of doubling the healing rate of a full-thickness defect using combinations of three treatments, for the first time, each shown to increase healing rate: application of transforming growth factor-β_3_ (TGF-β_3_), an albumin based scaffold, and mesenchymal stem cells (MSCs). At one week following surgery, the combined treatment showed the greatest increase in healing rate, particularly for the epithelialization rate. Although the target level of a 100% increase in healing rate over the control was not quite achieved, it is anticipated that the goal would be met with further optimization of the treatment.

## 1. Introduction

Skin is the largest organ in the body and it has a variety of functions; mostly protection of underlying tissue and organs. Pressure ulcers are one of the most common forms of skin injury, creating open wounds, and they are difficult to heal as well as costly to the patient and the overall health care system. They occur primarily when skin covering a bony prominence, such as the sacrum, is compressed for an extended period resulting in tissue necrosis.

Patients with spinal cord injury (SCI) are particularly susceptible to pressure ulcers due to limited mobility and the lack of sensation in the area caused by injury to the nervous system. Between 20–30% of SCI patients, 10% of all hospitalized patients, and 5% of community living patients develop pressure ulcers [1,2,3]. In the United States (U.S.), there are about 2.5 million patients with pressure ulcers per year at a cost of about $10 billion/year; with a pressure ulcer increasing the average cost of just a hospital stay by over $40,000 [4]. About 60,000 patients die each year due to complications of pressure ulcers [4].

Pressure ulcers can be different depths with the full-thickness ones (Stage III and IV) responsible for most of the increased cost. Although a problem for all populations, these pressure ulcers are more difficult to heal with SCI patients; since bed rest is typically needed. Prolonged bed rest, in this population, not only interferes with mobility, but it causes delays in achieving rehabilitation goals, reduced educational opportunities, and the subsequent long-term impact on vocational potential, separation from the family unit with its impact on psychological and social development, and the loss of general personal independence and productivity that contribute to self-esteem. The cost is therefore more than just increased hospital costs, rehabilitation costs, and loss of income [1,2,3,4,5]. 

There are currently two main treatment options for (Stage III and IV) SCI pressure ulcer patients: non-surgical treatment requiring roughly six months of bed rest and continual dressing changes or surgical treatment (most commonly skin flap surgery) requiring roughly six weeks of bed rest [5]. Though surgical treatment typically produces faster healing; there is still a significant amount of bed rest required, and the recurrence rates are high [5].

Surgical treatment is also costly adding over $40,000 to total costs [6]. Non-surgical treatments, however, with additional bed rest can have significantly more costs; $6000/day in-patient and about half of that at home [7]. 

Therefore, there is a need to develop a non-surgical treatment that speeds healing to a point close to that of surgical treatment without significant cost inflation to efficiently return the patient to a more normal quality of life without financial burden to the family and/or health care providers.

In order to approximate the healing time of surgical treatments, a non-surgical treatment would need to reduce healing time by about half (i.e., increase healing rate by a factor of two). Current non-surgical treatments include the use of occlusive dressings, such as polymer films, hydrogels, and hydrocolloids to provide a protective barrier to the wound while preventing water loss [8,9,10,11]. Other non-surgical treatments include the application of topical agents, such as growth factors and disinfectants to improve healing as well as cell-based therapies [8,9,10,11,12,13,14,15,16,17,18,19,20,21]. Though these approaches have boosted healing, they still fall short of surgical treatment, indicating the need for new more effective non-surgical treatments.

There are currently few clinically approved active treatments to speed healing in skin wounds with most lacking FDA approval for pressure ulcers [8,9]. There are three major components of healing that could be enhanced as part of an active treatment: cells to reconstitute the wound bed, a matrix to provide structural support for cells reconstituting the wound, and growth factors to orchestrate the healing process. By providing these three elements, it may be possible to increase healing rates closer to those seen with surgical treatment.

The goal of this study was to evaluate a treatment for pressure ulcers that addressed the different aspects of the wound healing process by combining three proven treatments: biodegradable albumin scaffold, mesenchymal stem cells (MSCs), and transforming growth factor-β_3_ (TGF-β_3_). It was anticipated that by combining a degradable albumin scaffold with MSCs treated ex vivo to overexpress TGF-β_3_ seeded within the scaffold and injected along the wound edges the treatments would work synergistically to increase the healing rate by two times that of the control. These combined treatments were evaluated in a full-thickness wound rabbit model for one and two-week time periods in this study.

This is a feasibility study to determine if it is possible to double the healing rate using a degradable regenerative scaffold with and without added bioactivity (stem cells and a growth factor). A full-thickness wound will be used, vs. a wound caused by pressure. Once a full-thickness pressure ulcer (Stage III or IV) is debrided and the pressure is removed, the wound acts similar to a full-thickness wound with little underlying pathology and treatments being developed in this model worked similarly clinically for pressure ulcers [22]. 

Albumin was selected as the degradable tissue adhesive scaffolds based on previous studies [23,24,25,26,27,28]. This includes studies to optimize the concentration of the components and the crosslinker (one included in this issue), to use for full-thickness wounds, to obtain the desired degradation profile and mechanical properties over time [24,25,26]. Studies have also been done to optimize cell seeding and growth factor delivery [23,24,25,26,27,29]. Albumin is inexpensive and it is easier to remove contaminants than for other serum proteins used for degradable regenerative tissue adhesive scaffolds, such as fibrinogen [26,27,28,29,30].

The scaffold serves to enhance the speed of healing by allowing cells to be seeded into or grow into the biomaterial and produce ECM (extracellular matrix) versus having to produce ECM first before cells can grow into the wound [17,29]. This is particularly beneficial when the increased cell proliferation and migration is induced by added biologic factors [23,29]. Using a tissue adhesive allows for the scaffold to be formed in situ conforming to and attaching to the wound versus having to be cut and sutured in place; making it more amenable to treatment at the patient’s home [27,28].

Mesenchymal stem cells (MSCs) were selected for the promise that they have shown in many areas of tissue engineering and regenerative medicine [8,16,18,31,32,33,34,35,36,37,38,39,40,41,42,43,44,45,46,47]. Although only a small fraction of the bone marrow cells, they home to wounds [38]. Specific studies have shown MSC’s role in reepithelialization [16,18,39], angiogenesis [16,32], ECM production [16,18], and overall healing [8,16,18,19,31,38,39].

TGF-β_3_ was selected for its regenerative ability; shown as a main reason why fetal wounds heal mostly by regeneration [48,49,50,51,52,53,54]. TGF-β_3_ has been shown to have a role in fibroblast migration, which allows for a more normal basketweave collagen organization; resulting in a reduction in scarring as well as improved healing [49]. This has been shown clinically for impaired wounds [48]. Both the timing of release and short in vivo half-life of TGF-β_3_ make it helpful for it to be released by cells growing into a healing wound [49,51]. In previous studies, the adenoviral vector containing the signal sequence, latency associated peptide, and C-terminus region of TGF-β_3_, along with a cytomegalovirus promoter were produced and the release kinetics and activity assays were done [51,53]. 

For commercializability, the added costs of the treatment (e.g., development cost and time, including regulatory approval) will need to be compared to the surgical option. This was partly taken into consideration by designing a system that could use autologous cells and be delivered at the patient’s home. The study was also designed to help determine if the added bioactivity was necessary to reach the clinical performance design constraint.

An additional design constraint is to have the increase in healing rate be due to regeneration vs. scarring. This is both an aesthetic and functional constraint [17]. For pressure ulcers, aesthetics is normally not a high priority, but recurrence is; with increased scarring leading to increased recurrence [17]. For other skin wounds, increased scarring typically reduces the aesthetics and is therefore is undesirable.

## 2. Materials and Methods

### 2.1. Preparation of Albumin Scaffolds

Albumin gels were made by mixing a solution of lyophilized albumin with a solution of poly(ethylene glycol) functionalized with disuccinimidyl glutarate at each end (PEG-SG_2_). The albumin solution was prepared within 24 h of use by gently dissolving fraction V lyophilized rabbit albumin (Sigma, St. Louis, MO) in a 0.9% saline solution at a concentration of 0.33 g albumin/mL. The solution was covered in foil and incubated at 4 °C while dissolving to prevent degradation. Once fully dissolved the albumin solution was sterile filtered through a 0.22 μm Milipore © syringe filter in a sterile environment and was stored at 4 °C until used.

The PEG-SG_2_ (Lakeshore Biomaterials, Birmingham, AL, USA) solution was prepared at the time of use by dissolving the PEG-SG_2_ powder in a basic N-2-Hydroxypiperazine-N′-2-ethanesulfonic acid (HEPES, Fisher Scientific, Fairlawn, NJ, USA) buffer solution. The buffer solution was made by mixing 11.93 g HEPES, 7.03 g NaCl, 0.38 g KCl, and 0.432 g MgCl_2_·6H_2_O with distilled water and altering the pH to 9.2 with NaOH. The HEPES buffer solution was filtered through a Millipore © syringe filter in a sterile environment and stored at room temperature until used. 

#### 2.1.1. Mesenchymal Stem Cell Isolation and Characterization

As done previously [51,53], bone marrow mononuclear cells were separated by centrifugation in a Ficoll–Hypaque gradient (density 1.077 g/cm^2^; Sigma, St Louis, MO, USA), washed twice with Hanks’ balanced saline solution (HBSS), and suspended in Dulbecco’s minimum essential medium-F12 50/50 (DMEM-F12 50/50, Sigma, St Louis, MO, USA) containing 10% fetal calf serum (FCS; Gibco BRL, Grand Island, NY, USA). Hematopoietic cells and non-adherent cells were removed by washing the culture the next day, followed with changes in medium. Adherent cells after the second subculture, referred to as second generation MSCs, were used to prepare frozen stocks.

Chondrogenic, adipogenic, and osteogenic differentiation assays were performed to ensure that the cells had the characteristics of MSCs. These samples were used to initiate the MSC culture for in vivo studies.

#### 2.1.2. Fluorescent Labeling of Mesenchymal Stem Cells

All cells used were labeled 24 h prior to surgery with chloromethylbenzamido (CM-DiI, Invitrogen, Carlsbad, CA, USA). The stock solution prepared at 10 mM concentration in dimethylsulfoxide was diluted in Dulbecco’s phosphate buffered saline (PBS, Sigma, St. Louis, MO, USA) to a working concentration of 2 μM. Cells plated in T-75 flasks had medium aspirated and replaced with 7 mL of the working solution of CM-DiI. The flasks were incubated for 5 min at 37 °C and then for 15 min at 4 °C to decrease incorporation into cytoplasmic vesicles. The solution was then aspirated from the flasks, the cells washed twice with PBS, and then incubated at 37 °C in DMEM-F12 50/50.

#### 2.1.3. MSC Transduction and Preparation

Based on previous studies [51,53], twenty-four hours prior to surgery MSCs were transduced with AdRGDpK7TGFβ_3_ (2 × 10^12^ vp/mL). Two T-75 flasks that were previously seeded with MSCs were trypsinized and counted to enable an accurate multiplicity of infection (MOI). The cells were then re-seeded onto the flasks and allowed to re-adhere. Once adherent the medium was aspirated from the flask and replaced with 7 mL of DMEM-F12 50/50 containing 2% FBS and AdRGDpK7TGFβ_3_ in a concentration such that the MOI was 5000. The flasks were then incubated for 2 h at 37 °C before removing the transduction medium and replaced with 15 mL of 10% FBS in DMEM-F12 50/50. The transduced cells were incubated overnight at 37 °C until the time of surgery.

Prior to surgery transduced MSCs (MSC-TGFβ_3_s) and untreated MSCs were trypsinized. MSC-TGFβ_3_s and untreated MSCs that were to be injected were suspended in 1 mL of sterile PBS at a concentration of 4 × 10^5^ cells/mL. Cells that were to be incorporated into the scaffold were suspended in the 0.33 g/mL albumin solution at a concentration of 4 × 10^5^ cells per 0.2 mL of albumin solution. The samples were placed on ice until used.

### 2.2. Animal Surgery

Eight female New Zealand White rabbits weighing approximately 3 kg were used in the study (Brown Family Farms, Trussville, AL, USA). Each received six wounds with one wound serving as control. The animals were studied at one or two weeks post-wounding, with four animals in each time period. 

**Figure 1:**
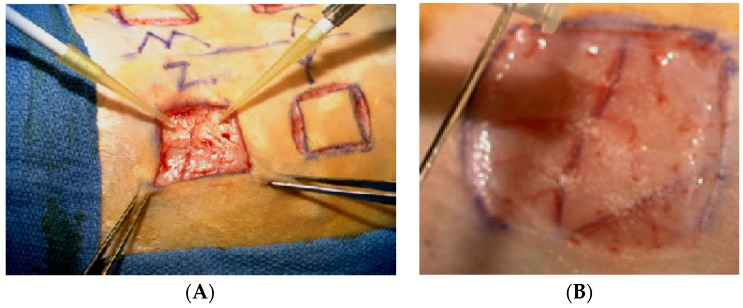
Full-thickness wound (**A**) adding the albumin scaffold (**B**) after scaffold crosslinking.

For surgery the animals were anesthetized with ketamine and xylazine administered intramuscularly and supplemented with isoflurane. Six 2 cm × 2 cm full thickness wounds extending down to the panniculus carnosum muscle were placed on the dorsum using a scalpel (Figure 1A). Gauze soaked in sterile saline was placed on the wounds prior to treatment to prevent desiccation.

The wounds were treated as follows: no treatment (control), albumin scaffold (Figure 1) untreated MSCs injected at all four wound edges (4 × 10^5^ cells) (MSC), MSCs transduced with Ad-TGFβ_3_ injected at all four wound edges (4 × 10^5^ cells/mL) (MSC-TGFβ_3_), albumin scaffold seeded with untreated MSCs (4 × 10^5^ cells), and untreated MSCs injected at four wound edges (4 × 10^5^ cells) (MSC-A), and albumin scaffold seeded with MSCs transduced with Ad-TGFβ_3_ (4 × 10^5^ cells) and MSCs transduced with TGFβ_3_ injected at four wound edges (4 × 10^5^ cells/mL) (MSC-TGFβ_3_-A).

For wounds receiving albumin scaffolds two syringes were filled with the PEG-SG_2_ solution and the albumin solution (or albumin + cells) and were applied sequentially to the wound with 0.2 mL of each solution used. The corners of the wound were elevated using Allis clamps until the initially low viscosity scaffold cured. During curing the Allis clamps were maneuvered to mix the scaffold components as well as facilitate an even distribution of cells. Wounds receiving injections of cells had 0.25 mL of cells suspended at 4 × 10^5^ cells/mL injected along each edge. The needle was inserted at one vertex of the wound in the outer intact dermis and was pushed through the dermis of the wound edge until the tip of the needle resided at the next vertex. The needle was then slowly pulled back through the dermis while pushing gently on the plunger to deliver an even distribution of 0.25 mL cell suspension per wound edge.

The wounds were then covered with an occlusive dressing (Tegaderm, Cardinal Health, Dublin, OH, USA) to prevent infection and to keep the wounds moist. Rabbit jackets and stockinettes were used to prevent the rabbits from irritating the wounds or removing the dressings. The rabbits were then observed until awake and alert and returned to the cage.

At the selected time period the animals were euthanized with an overdose of pentobarbital. The wounds were measured, removed, and fixed in 10% neutral buffered formalin. The wounds were then bisected down the midline, embedded in paraffin, and sectioned along the bisected edge. The tissues were stained with hematoxylin and eosin as well as left unstained for observation of fluorescently labeled cells.

### 2.3. Histomorphometry

**Figure 2:**
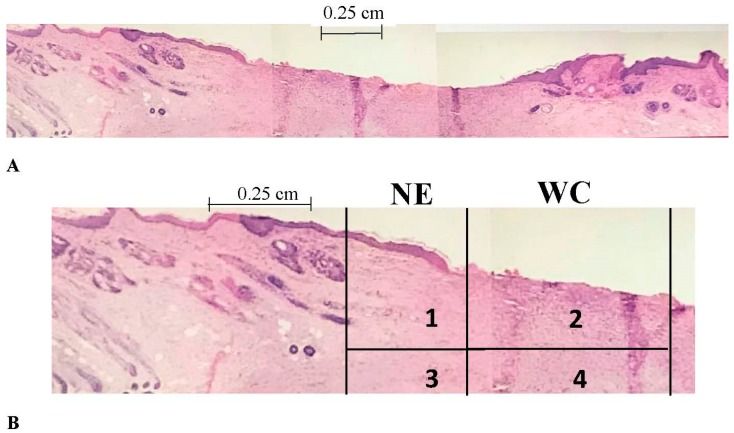
(**A**) A histology slide of the entire wound surrounded by the original skin; (**B**) Part of the same image with critical areas labeled. Area 1 and 3 are under the new epithelium (NE) and would be the outer part of the wound. The areas in the wound center (areas 2 and 4) would be considered the middle of the wound.

Histomorphometry is a method used to quantitatively determine the 3-D microscopic structure of tissues from two-dimensional images. It enables a quantitative measure of the desired healing parameters as described below. All images were taken using a Nikon SMZ 1000 microscope (Nikon Instruments Inc., Melville, NY, USA) with an attached Spot Insight Color Camera (Spot Imaging Solutions, Sterling Heights, MI, USA). Epithelialization and contraction were measured using Spot Advanced Software (Spot Imaging Solutions, Sterling Heights, MI, USA). Figure 2 shows a representative wound as well as identifies the areas that were examined.

#### 2.3.1. Epithelialization Rate

Epithelialization rate (ER) was used to determine how quickly new epithelial tissue grows from the edge of the wound. The average lengths of new epithelial layers from each wound margin (area 1 in Figure 2B) were determined and the ER was calculated using the equation below, where *t* is the time in weeks. The length of epithelium in area 1 (Figure 2B) is *EL*_1_ with *EL*_2_ the same measurement on the other side of the wound.

$$ER=\frac{EL_{1}+EL_{2}}{2t}$$

#### 2.3.2. Contraction Rate 

Contraction rate (CR) represents the change in wound size from the original per unit time. The original wound width, *W*_o_, determined at the time of surgery was used with the new wound width, *W*_n_, determined from histology slides and measurements at sacrifice in the equation below. The values that were obtained from histology were compared to the measurements at sacrifice to ensure accuracy. *W*_n_ is the length from the left edge of area 1 (Figure 2B) to the right edge of the corresponding area on the other side of the wound (seen in Figure 2A).

$$CR=\frac{Wo-Wn}{2t}$$

#### 2.3.3. Healing Rate

Overall healing rate (*HR*) was determined by adding *ER* and *CR*.

*HR* = *ER* + *CR*

#### 2.3.4. Epithelialization Rate/Contraction Rate Ratio

In order to obtain a measure of regenerative healing versus scarring the ratio of epithelialization rate (more indicative of regenerative healing) and contraction rate (more indicative of scarring) was calculated. 

#### 2.3.5. Fluorescent Cell Volume Fraction

**Figure 3:**
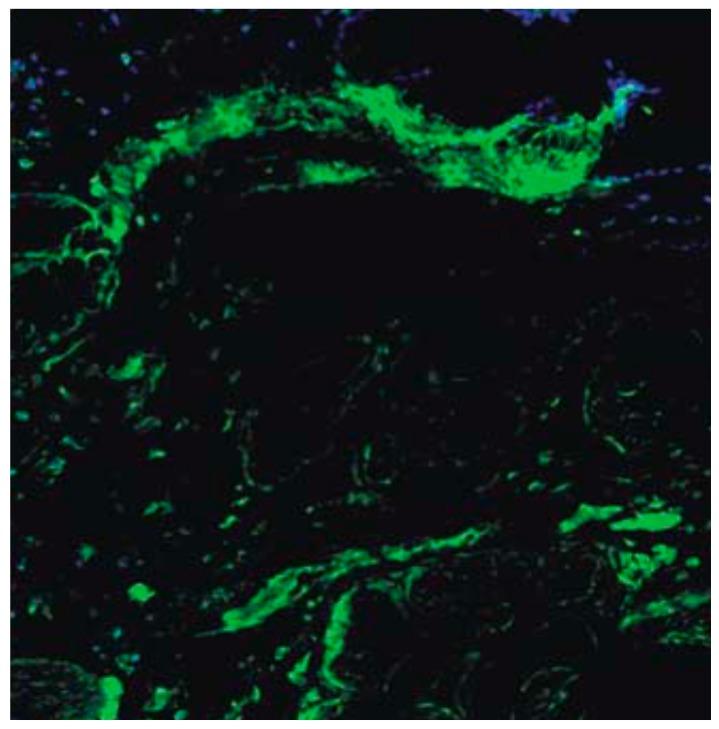
Imaging of mesenchymal stem cells (MSCs) transplanted in the wounds. Shown is a representative fluorescence microscopy image of MSCs (green) after three weeks in vivo.

In order to determine the presence and quantity of fluorescently labeled cells at sacrifice, unstained sections were viewed under a fluorescent microscope capable of digital image capture (Figure 3). The image files were opened in Adobe Photoshop and overlaid with a 165 point grid. The number of cells present at intersections of the grid was divided by the total number of intersections to give a volume fraction of labeled cells. This volume fraction approximates the volume fraction of cells in three-dimensions. The treatment group of the slide was blinded to the observer. Two random images from each sample were taken from within the wound margins (inner cell volume fraction—areas 2 and 4 in Figure 2B), and from each wound edge (outer cell volume fraction—areas 1 and 3 in Figure 2B). An in vitro model was used to approximate the volume fraction within the scaffold immediately following scaffold application to investigate changes in inner volume fraction from *t* = 0 to sacrifice. A well was constructed using pieces of tissue culture plastic cut from T-75 flasks that were glued to the bottom of a six-well plate to form a square with 2 cm × 2 cm dimensions to approximate the wound. Scaffold volume and the number of cells seeded were the same as those used in the animal model. Once the scaffold cured, the apparatus was immediately imaged under a fluorescent microscope. Four random images were taken.

$$Volume\;Fraction=\frac{\#\;Cells}{\#\;\mathrm{Intersections}}$$

### 2.4. Statistical Analysis

Results from treated wounds were compared with results from the control on the same animal using a paired *t*-test. Analysis of variance (ANOVA) and post hoc Tukey’s pairwise comparisons were also used to compare between treatments and weeks. The study was designed with every animal receiving every treatment so that each treatment can be compared to the control using a paired *t*-test. With significance set at α = 0.05, an appropriate statistical power (1-β, where β = 0.5) of 0.5 for the paired *t*-test, and an estimated ratio of the change in means to the standard deviation of 1.35 an *n* = 4 was adequate in detecting changes in healing parameters of 100%.

## 3. Results

### 3.1. Epithelialization Rate

**Figure 4:**
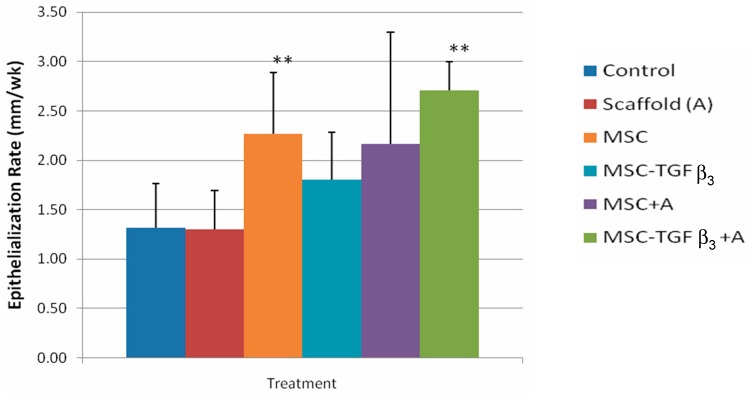
Epithelialization rates at 1 week following surgery. ** indicates statistical significance between treatment and control. * indicates statistical significance between two treatments (*p* < 0.05).

Figure 4 shows ER at 1 week following surgery. At one week following surgery wounds treated with MSC-TGFβ_3_-A showed a significant increase in ER compared to the control (105.6% increase, *p* = 0.0009). Wounds treated with MSCs injected along the wound edges also showed a significant increase in ER as compared to the control (71.9% increase, *p* = 0.02).

**Figure 5:**
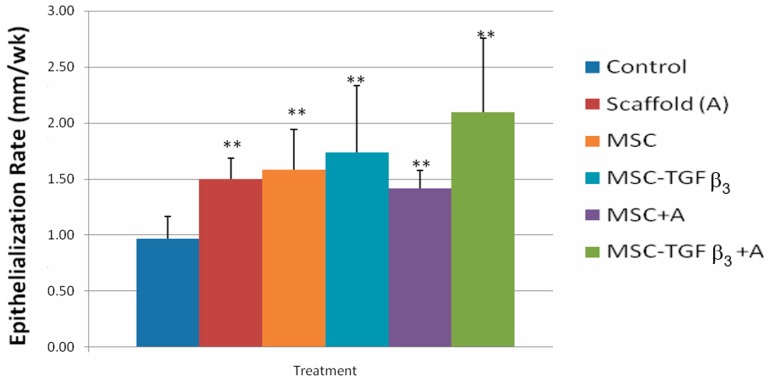
Epithelialization rates two weeks following surgery. ** indicates statistical significance between treatment and control. * indicates statistical significance between two treatments (*p* < 0.05).

Figure 5 shows ER at two weeks following surgery. Wounds treated with MSC-TGFβ_3_-A showed the greatest increase over the control (117% increase, *p* = 0.008). Wounds treated with A (scaffold only) (55%, *p* = 0.004), MSC-A (47%, *p* = 0.005), MSC (64%, *p* = 0.01), and MSC-TGFβ_3_ (80%, *p* = 0.02) all had significant increases in ER as compared to the control. No other statistically significant differences in ER were observed between the treatment groups.

### 3.2. Contraction Rate

Table 1 shows the values obtained for CR at one and two weeks following surgery. No treatment showed a significant increase or decrease in CR compared to the control at either week.

### 3.3. Healing Rate

**Figure 6:**
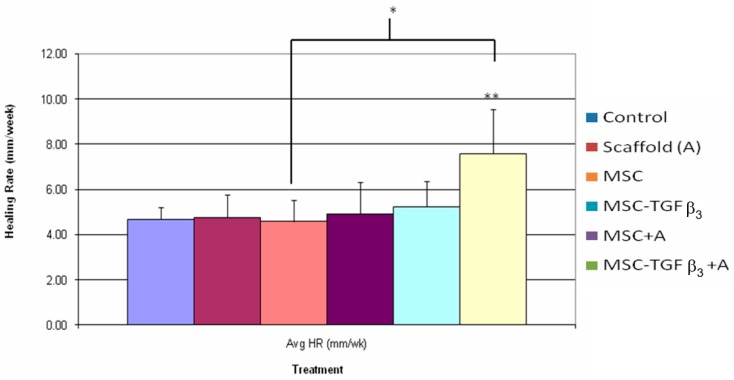
Healing rates one week following surgery. ** indicates statistical significance between treatment and control. * indicates statistical significance between two treatments (*p* < 0.05).

Figure 6 shows HR at one week following surgery. Wounds treated with MSC-TGFβ_3_-A were the only treatment to show a statistically significant difference compared to the control, with MSC-TGFβ_3_-A treated wounds showing an increase as compared to the control (62% increase, *p* = 0.05). MSC-TGFβ_3_-A treated wounds were also statistically higher than MSC treated wounds (65% increase, *p* = 0.04).

**Figure 7:**
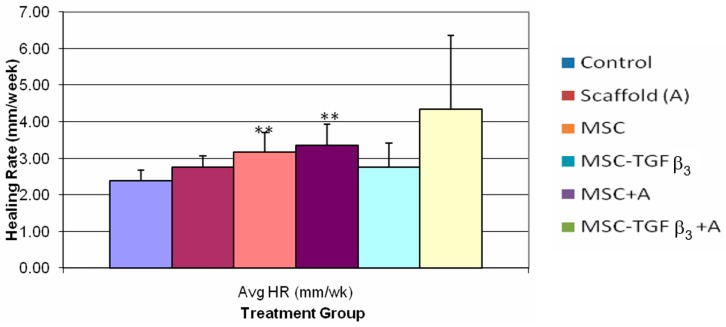
Healing rates two weeks post-surgery. ** indicates statistical significance between treatment and control (*p* < 0.05).

Figure 7 shows HR at two weeks following surgery. Wounds that were treated with MSC-TGFβ_3_ had significant increase in HR as compared to the control (40% increase, *p* = 0.02). MSC treated wounds were also significantly higher than the control (32% increase, *p* = 0.02). No other statistically significant results were observed.

### 3.4. Epithelialization Rate/Contraction Rate Ratio

**Figure 8:**
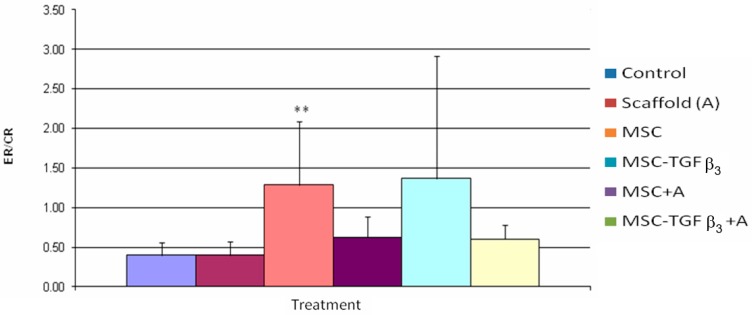
Epithelialization rate/contraction rate (ER/CR) ratios one week following surgery. ** indicates statistical significance between treatment and control (*p* < 0.05).

Figure 8 shows the ER/CR ratio one week following surgery. MSC treated wounds were the only wounds to show a significant increase in ER/CR ratio (221% increase, *p*= 0.04). No statistically significant differences were observed among the treatment groups.

Table 2 shows the ER/CR ratio two weeks following surgery. There were no statistically significant differences between any treatment and the control, and none found among the treatment groups.

### 3.5. Fluorescent Cell Volume Fraction

**Figure 9:**
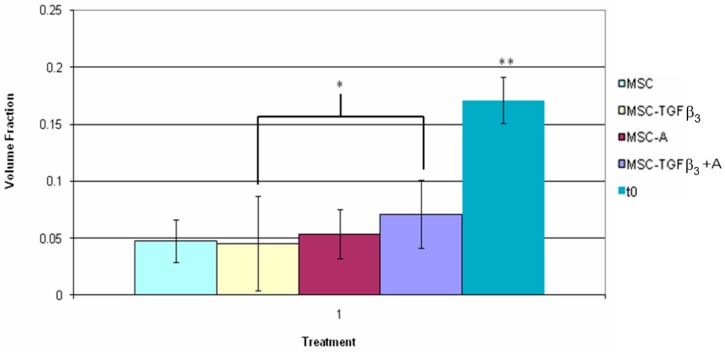
Inner fluorescent cell volume fraction one week following surgery. * indicates statistical significance between two treatments, and ** indicates a significant difference with all other treatments (*p* < 0.05).

Figure 9 shows the volume fraction of fluorescent cells found between the wound edges 1 week following surgery as well as the in vitro assessment of volume fraction within the scaffold immediately after seeding. MSC-TGFβ_3_-A treated wounds showed a greater volume fraction of fluorescent cells within the wound bed than MSC-TGFβ_3_ treated wounds (57% increase, *p* = 0.02). Each treatment group showed a significant decrease in volume fraction as compared to the in vitro scaffold. MSC-TGFβ_3_ treated wounds had a decrease in volume fraction of 73% (*p* = 0.0001) when compared to the in vitro model. MSC treated wounds had a similar decrease of 72% (*p* = 0.001). MSC-A treated wounds followed with a decrease of 69% (*p* = 0.0001), and MSC-TGFβ_3_-A treated wounds had the smallest decrease in fluorescent cell volume fraction (58%, *p* = 0.0001) compared to the in vitro model.

**Figure 10:**
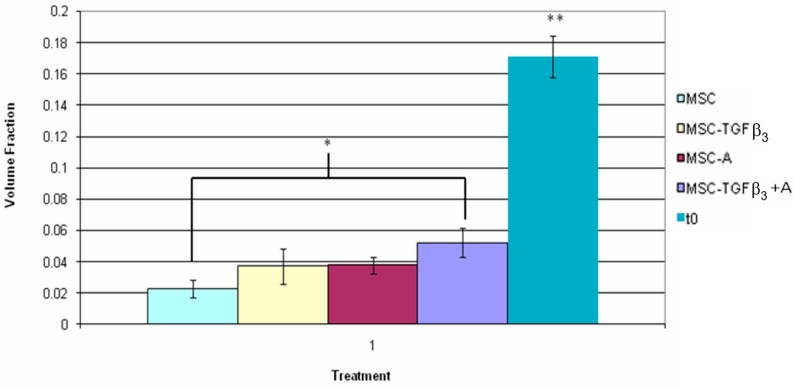
Inner fluorescent cell volume fraction two weeks following surgery. * indicates statistical significance between two treatments, and ** indicates a significant difference with all other treatments (*p* < 0.05).

Figure 10 shows the volume fraction of fluorescent cells found between the wound edges 2 weeks following surgery as well as the in vitro assessment of volume fraction within the scaffold immediately after seeding. MSC-TGFβ_3_-A treated wounds showed a greater number of fluorescently labeled cells than MSC treated wounds (78% increase, *p* = 0.001). At 2 weeks all groups also showed a significant decrease in volume fraction as compared to the in vitro scaffold. MSC treated wounds had a decrease of 87% (*p* = 0.0001) in fluorescent cells found within the wound edges, followed by MSC-TGFβ_3_ treated wounds (78%, *p* = 0.0001), MSC-A treated wounds (77%, *p* = 0.0001), and MSC-TGFβ_3_-A treated wounds (69%, *p* = 0.0001).

**Figure 11:**
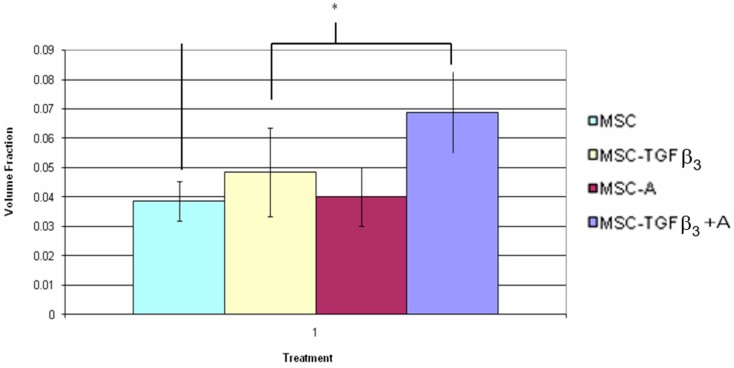
Outer fluorescent cell volume fraction two weeks following surgery. * indicates statistical significance between two treatments (*p* < 0.05).

Table 3 shows the results of the volume fraction of fluorescent cells found within the wound edges one week following surgery. At this time point, no significant differences were observed. Figure 11 shows the volume fraction of fluorescent cells found within the wound edges two weeks following surgery. Wounds treated with MSC-TGFβ_3_-A showed a significant increase in fluorescent cells compared to MSC-A treated wounds (78% increase, *p* = 0.02) as well as MSC treated wounds (71% increase, *p* = 0.015).

## 4. Discussion

The overall goal of the study was to evaluate the effectiveness of a combinational treatment for full-thickness wounds consisting of a biodegradable albumin scaffold, mesenchymal stem cells, and the overexpression of TGF-β_3_. This was done in an animal model to determine the feasibility of using the treatments alone or in combinations in a degradable regenerative system to meet the clinical performance design constraints; a HR of at least two times that of the control, which would approximate the healing rate of surgical treatment. The goal was to see how close the current system was to the desired clinical performance. Although not a pressure induced wound in a spinal cord injured animal, it will help to decide strategies that should be effective clinically. It will be important to more fully determine the mechanism of any benefits seen to determine where further optimization can be done in vivo before beginning clinical studies. For example, the cell-matrix interactions have not been optimized to promote MSC viability and proliferation [54]. Further, the amount of TGFβ_3_ release has not been optimized yet. Also, a model that is more specific to pressure ulcers and requiring more than 2–4 weeks to heal needs to be explored.

Healing (skin wound closure) occurs through two processes—epithelialization and contraction—which, when added, give the overall healing rate [17]. Epithelialization is the process of covering the wound with new tissue resulting in regeneration of the skin, while contraction is the pulling together of the wound edges and is essentially a scarring process [17]. As such, wounds can have the same healing rate, but different outcomes in terms of quality (aesthetics and function). It was therefore desired that the treatment provides an increase in HR by increasing ER and CR, while maintaining or increasing an ER/CR ratio relative to the control. Maintaining this ratio indicates that the treatment provides the same quality of healing in terms of scarring as the control. It was hypothesized that the combination treatment would produce a higher healing rate than the control as well as each of the treatments individually.

As hypothesized wounds treated with MSC-TGFβ_3_-A showed a statistically higher HR at 1 week than the control. None of the other treatment groups achieved a significant difference from the control at one week. The increase in HR induced by MSC-TGFβ_3_-A was 61%, which is clinically significant but short of the goal of 100%. This indicates that a wound healing in 6 months with standard treatment would heal in 3.66 months (versus the target of three months) with the combinational treatment. MSC-TGFβ_3_-A treated wounds also had a statistically higher HR than MSC treated wounds alone with an increase of 66%.

At two weeks wounds treated with MSC and MSC-TGFβ_3_ were the only wounds to show a significant increase in HR as compared to the control (40% and 32%, respectively.) Although MSC-TGFβ_3_-A treated wounds were highest on average (81% increase), it appears that one low HR result led to a high enough standard deviation to make the difference non-statistically significant when compared to the control (*p* = 0.06). In order for the test to be sensitive enough to detect this difference with a statistical power of 0.50 (typical *t*-test level), a sample size of 8 would have been necessary.

When the healing rate was broken down into its two components, statistical differences in ER, but not CR, were observed. MSC-TGFβ_3_-A treated wounds had the highest increase in ER overall at one week, with a statistically significant increase of 106% compared to the control. Wounds treated with MSC were also statistically higher than the control with an increase of 72%. Wounds treated with MSC-TGFβ_3_, however, had about half of that increase (37% increase) although it was not a statistically significant difference (*p* = 0.09). At two weeks, all treatment groups had a statistically higher ER than the control. MSC-TGFβ_3_-A treated wounds produced the highest increase compared to the ER of the control (117%), but no statistical differences were found among treatment groups. At one week, no treatments showed a statistically significant increase or decrease in CR when compared to the control. MSC-TGFβ_3_-A treated wounds were higher on average, but the results were not statistically significant (*p* = 0.075, 44.90% increase). Wounds treated with MSC produced the only lower average CR than the control, but again the results were also not statistically significant (*p* = 0.07, 31% decrease). At two weeks, there were no statistically significant differences in CR.

To further analyze the quality of healing the ER/CR ratio was calculated; with the higher the ratio the more of the healing was regenerative. The percent regenerative healing can be approximated by the ratio/(ratio + 1).

At one week, MSC treated wounds were the only wounds to show a statistically significant increase in ER/CR ratio (221% increase or from about 30% regenerative healing to about 55%). Wounds treated with MSC-TGFβ_3_-A and MSC-TGFβ_3_ had a higher ER/CR ratio than the control, but the results were not statistically significant (*p* = 0.07, 51% increase and *p* = 0.09, 57% increase, respectively). At two weeks, there were no differences in ER/CR ratios, though wounds treated with A was close to being statistically significant (*p* = 0.051, 62% increase).

Although the critical clinical parameters are the healing rates and ratios, it is helpful to understand the mechanism. Future studies will look at the cells and blood vessels present at specific time periods. In this study, only the fate of the stem cells was examined. There is concern that the signal fades over time particularly due to cell mitosis; meaning the data comparing time periods may not be as reliable as the ones comparing treatments at the same time period.

To compare the ability of the different systems to maintain the presence of seeded and injected cells, the volume fraction of fluorescent cells was calculated. At one week, the only difference among the treatment groups came from the inner fluorescent cell volume fraction of wounds treated with MSC-TGFβ_3_-A and MSC-TGFβ_3_. MSC-TGFβ_3_-A treated wounds had 57% more fluorescent cell volume fraction within the wound edges than MSC-TGFβ_3_ alone treated wounds, as well as the highest volume fraction of cells within the wound edges of all groups (7% total volume fraction). With more MSCs overexpressing TGF-β_3_ and taking part in the healing process from within the wound bed, the combined treatment had a greater opportunity to increase the overall healing rate.

The outer fluorescent cell volume fraction at one week showed no significant differences among any of the groups as expected, since each group received injections of cells along the wound edges. At two weeks, MSC-TGFβ_3_-A treated wounds again had the highest inner fluorescent cell volume fraction (5% total volume fraction), but a statistically significant difference was only found with MSC treated wounds (78% more inner fluorescent cell volume fraction). At two weeks, MSC-TGFβ_3_-A treated wounds maintained the highest outer fluorescent cell volume fraction (7% total volume fraction), and also was significantly greater than wounds treated with MSC-A and MSC (78% and 72% more fluorescent cell volume fraction, respectively).

When compared to the in vitro scaffold at *t* = 0, all treatments at each time point showed a significant decrease when examining the inner fluorescent cell volume fraction (corresponding to where the scaffold was placed). At one week, wounds treated with MSC-TGFβ_3_-A showed the lowest decrease (54% decrease compared to the in vitro model), followed by wounds treated with MSC-A (67% decrease as compared to in vitro model), MSC (71% decrease compared to the in vitro model), and MSC-TGFβ_3_ (72% decrease compared to the in vitro model). As was expected, the treatments containing the scaffold seeded with cells had the highest volume fraction of cells. 

Badiavas et al. attempted to use MSCs in the treatment of chronic wounds, but found difficulty in delivering and maintaining cells within the wound bed [20]. To prevent this complication, the albumin scaffold was used to provide a structural entity with which the implanted cells could adhere as well as a substrate for cells migrating from the wound edges; although it can be further optimized [54]. At two weeks, MSC-TGFβ_3_-A treated wounds again had the lowest decrease in inner fluorescent cell volume when compared to *t* = 0 in the vitro model (58% decrease), followed by wounds treated with MSC-TGFβ_3_ (70% decrease), MSC-A (75% decrease), and MSC (76% decrease). 

By the end of week 2, the scaffolds had been biodegraded, necessitating the need for the regenerated tissue to be the scaffold. Therefore, the better regenerative scaffolds probably had the best chance of retaining implanted cells. The decrease in cells from the in vitro control could be attributed to many factors. During the creation of wounds, though great care was taken, small cuts occurred exposing the underlying muscle layer. Prior to the scaffold curing seeded cells may have leaked into the cuts decreasing the cells that were located within the wound. Migration of implanted cells to the surrounding undamaged tissue both beside and under the wound may also have occurred. Cells may have also been damaged during the period between cell preparation and application to the wound. At both time points, it was noted that some fluorescent cells were present in the inner layer of blood vessels, suggesting the possible differentiation into endothelial-like cells. This was expected based on the evidence provided by Wu, et al. that showed MSCs assist in healing through differentiation into a number of cells, including endothelial and epithelial like cells, as well as angiogenesis [16].

At 1 week MSC-TGFβ_3_-A treated wounds produced the highest increase in HR compared to the control. When broken down the HR shows that MSC-TGFβ_3_-A accomplished this by increasing the ER significantly as compared to the control as desired, but it also increased the CR on average (though not significantly). If the combined treatment had not increased CR compared to the control the HR would have only been roughly 30% greater. Because the treatment also increased CR, the ER/CR ratio was not significantly different, indicating that the quality of healing was about the same as the control in terms of scarring. By providing a significant increase in ER and slight increase in CR, the combined treatment achieved a greater HR than the control resulting in the best treatment at one week as hypothesized. MSC was the only treatment to produce a significant decrease in the ER/CR ratio indicating a more regenerative healing process, but it did not produce an overall HR significantly greater than the control. At two weeks, MSC-TGFβ_3_-A still produced the highest HR on average, but it was not statistically significant when compared to the control. ER continued to be significantly higher than the control, but increases in CR diminished as it did in all groups decreasing the overall HR. MSC and MSC-TGFβ_3_ were the only treatments that caused a significant increase in HR at two weeks. At this time point, it appears that the positive impact of the scaffold may have decreased (unless a scaffold is regenerated) since it has degraded. To maintain a consistent significant increase in HR at two weeks and beyond re-application of the scaffold may be required.

An explanation of the success of MSC-TGFβ_3_-A could be attributed to the synergistic manner in which the different facets of the treatment acted. The albumin scaffold provided a provisional matrix for cells taking part in epithelialization, thus boosting ER. TGF-β_3_, a growth factor that causes fibroblast migration across the wound bed, overexpressed by the implanted MSCs may have increased the number of fibroblasts in the wound bed, which could have then differentiated into myofibroblasts, causing increased wound contraction while also causing collagen to be deposited in a more organized fashion, allowing for better control of scar-inducing tension as indicated by Ferguson, et al. [14]. From the data, it appears that the primary mode of action was increasing ER, though non-significant increases in CR were observed. The dramatic increase in ER was expected based on previous results found when using MSCs or TGF-β_3_. In early investigations of TGF-β_3_, Ferguson et al. found that by increasing levels of TGF-β_3_ a higher level of regeneration, or re-epithelialization, could be achieved [14,21]. In other studies, cells that were genetically modified to overproduce TGFβ_3_ have led to a significant improvement in incisional wound healing [17,22].

In multiple studies using MSCs as a treatment for a variety of wounds, investigators found that MSCs also promote regeneration of the epithelial layer, which was observed in the current study [18,19,23]. MSCs also help mediate the healing process by producing factors that recruit cells to the wound bed and promote healing [23]. 

Overexpression of TGF-β_3_ in conjunction with the production of other mediators by the MSCs may have aided in the increase in HR. MSC-TGFβ_3_-A also maintained the highest inner fluorescent cell volume at both time points, allowing the implanted cells to provide a bigger impact in terms of possible differentiation and the overexpression of TGF-β_3_ within the wound bed.

To elucidate the mechanisms behind the treatment’s success as well as further optimize the scaffolds system, further studies are needed. Both the fate of the MSC cells as well as their bioactivity (recruitment, proliferation, protein production, etc.) should be investigated.

All rabbits failed to remove the protective rabbit jacket and occlusive wound covering Tegaderm^®^ throughout the treatment. Though the coverings remained intact, the dressings were not changed between one and two weeks. At sacrifice, some of the wounds exhibited signs of desiccation, which would slow overall healing. This could explain the decrease in HR for some of the MSC-TGFβ_3_-A at two weeks as well as the slight increase in CR observed. Dressings were not changed due to the fear of removing the scaffold and implanted cells with the dressing. This dressing change and reapplying of the scaffold and treatments at week two, as the study seemed to suggest, may help this.

Future optimization studies will help to determine the appropriate interval for dressing changes and reapplication to help get closer to the clinical performance design constraint. In many chronic wounds the standard of care is bandage replacement at an interval of less than once a week. Some wounds may even require bandage changes every 6 h, depending on the circumstances. Frequent bandage changes are, however, usually due to excessive exudate and/or infection. Any scaffold system however, particularly for pressure ulcers, would require a reduction in bacteria level and/or debridement before use clinically. It is reasonable to design the system to last two weeks and apply it every two weeks until the wound heals. 

In addition, the actual effect on ER and CR clinically needs to be determined. The model is somewhat predictive [22], but rabbits typically heal more by contraction than would be seen clinically. For example, if the contraction rate clinically is half that in the rabbit model, with the epithelialization rate the same, the increase in HR would be close to 90% for both 1 and 2 weeks for the MSC-TGFβ_3_-A vs. the control. This would be close to the target levels vs. the 62% and 81% in the rabbit model.

Another design parameter that could be optimized is the level of TGF-β_3_. Throughout the study MSCs used to overexpress TGF-β_3_ were treated with a single MOI of AdRGDpK7TGFβ_3_. In vitro testing showed MSCs treated with 5000 MOI of AdRGFpK7TGFβ_3_ produced the growth factor at a concentration of roughly 0.50 ng/µL [51,53]. Though this is within the therapeutic range that is found in clinical studies [15], investigating higher MOIs may better improve the outcome of the combined treatment and cause an increase of HR closer to the 100% goal.

The desired HR for MSC-TGFβ_3_-A was two times that of the control making *n* = 4 adequate for detection of such a high increase. Since each treatment resulted in an increase in HR over the control, detecting a difference between the different facets of the treatment was more difficult. In order to determine which aspects of the combinational treatment produced statistically significant differences from each other, a larger number of animals is needed. Furthermore, future studies using different cell numbers will better illuminate the role of implanted cells in the healing process. These studies will provide a more concrete determination of the efficacy of the combinational therapy as compared to each aspect of the treatment. Again from a commercialization standpoint, the less added bioactivity required to meet the clinical performance design constraint the better. Also, depending on the cost of the treatment, an increase in healing rate less than the performance design constraint may still be a more desirable option than surgery for SCI pressure ulcer patients.

In conclusion, this study showed the feasibility of using this treatment to get close to the clinical performance design constraint. This study represents the first time all three healing strategies were used together and in combinations in a full thickness wound. The use of each individually and in combinations allowed for determining the benefits and contributions of each strategy. The combination of the albumin scaffold, MSCs, and overexpression of TGF-β_3_ had the greatest impact on HR as compared to the control at one week post-surgery. At 2 weeks post-surgery the combined treatment maintained the highest HR, but it was not statistically significant compared to the control. Further studies are needed with a larger sample size to determine the actual differences between treatment groups as well as to determine the mechanisms for improving healing to optimize the system.

There are a number of options to further optimize the biomaterial enhanced regeneration including: increasing cell number, amount of TGF-β_3_ overexpressed, the cell matrix interactions, and changing the dressing bi-weekly to prevent desiccation from interfering with healing to produce a greater increase in HR.

Again, this approach seems feasible as a commercial clinical treatment for pressure ulcers in SCI patients. There is still, however, the need to further optimize the treatment before deciding its viability as a commercial product, as well as the best way to use it clinically.

## Figures and Tables

**Table 1:** Contraction rates at one and two weeks following surgery.

	1 Week		2 Weeks	
	Avg CR (mm/wk)	SD CR	Avg CR (mm/wk)	SD CR
**Control**	3.37	0.43	1.42	0.45
**A**	3.47	0.92	1.25	0.25
**MSC-TGFβ_3_-A**	4.89	1.79	2.23	1.38
**MSC-A**	3.07	1.99	1.34	0.81
**MSC-TGFβ_3_**	3.11	1.10	1.62	0.55
**MSC**	2.31	1.19	1.58	0.18

**Table 2:** Values of ER/CR ratio at two weeks following surgery.

	2 Weeks	
	ER/CR	SD ER/CR
**Control**	0.77	0.38
**A**	1.24	0.31
**MSC-TGFβ_3_-A**	1.50	1.31
**MSC-A**	1.45	0.47
**MSC-TGFβ_3_**	1.23	0.63
**MSC**	1.00	0.14

**Table 3:** Values of outer cell fluorescent volume fraction 1 week following surgery.

	1 Week	
	Outer Fluor Volume Fraction	SD
**MSC-TGFβ_3_-A**	0.057	0.016
**MSC-A**	0.056	0.018
**MSC-TGFβ_3_**	0.050	0.035
**MSC**	0.061	0.012
